# The landscape of transcriptional profiles in human oocytes with different chromatin configurations

**DOI:** 10.1186/s13048-024-01431-2

**Published:** 2024-05-10

**Authors:** Yi-Ran Zhang, Ying Yin, Shi-Meng Guo, Yu-Fan Wang, Guang-Nian Zhao, Dong-Mei Ji, Li-Quan Zhou

**Affiliations:** 1 Institute of Reproductive Health/Center of Reproductive Medicine, Tongji Medical College, Huazhong University of Science and Technology, Wuhan, China; 2 Present Address: NHC Key Laboratory of Study on Abnormal Gametes and Reproductive Tract, Anhui Medical University, Anhui, China; 3 Department of Physiology, School of Basic Medicine, Tongji Medical College, Huazhong University of Science and Technology, Wuhan, China; 4 Hubei Key Laboratory of Drug Target Research and Pharmacodynamic Evaluation, Huazhong University of Science and Technology, Wuhan, Hubei China; 5 Department of Obstetrics and Gynecology, National Clinical Research Center for Obstetrics and Gynecology, Tongji Hospital, Tongji Medical College, Huazhong University of Science and Technology, Wuhan, China; 6 Key Laboratory of Cancer Invasion and Metastasis (Ministry of Education), Hubei Key Laboratory of Tumor Invasion and Metastasis, Tongji Hospital, Tongji Medical College, Huazhong University of Science and Technology, Wuhan, China

**Keywords:** Surrounded, Non-surrounded, Nucleolus, Transition, Oocyte

## Abstract

With increasingly used assisted reproductive technology (ART), the acquisition of high-quality oocytes and early embryos has become the focus of much attention. Studies in mice have found that the transition of chromatin conformation from non-surrounded nucleolus (NSN) to surrounded nucleolus (SN) is essential for oocyte maturation and early embryo development, and similar chromatin transition also exists in human oocytes. In this study, we collected human NSN and SN oocytes and investigated their transcriptome. The analysis of differentially expressed genes showed that epigenetic functions, cyclin-dependent kinases and transposable elements may play important roles in chromatin transition during human oocyte maturation. Our findings provide new insights into the molecular mechanism of NSN-to-SN transition of human oocyte and obtained new clues for improvement of oocyte in vitro maturation technique.

**Supplementary Information:**

The online version contains supplementary material available at 10.1186/s13048-024-01431-2.

## Background

Nowadays, the rate of infertility has greatly increased in a considerable number of couples [1–3]. In assisted reproduction technology (ART), screening high-quality oocytes is key to obtain early embryos with high developmental competency [1, 2]. With the help of maternal epigenetic regulators, parental chromatin gradually forms unique chromatin structures to activate the zygotic genome after fertilization [4, 5]. It can be concluded that the developmental quality of the oocyte affects the developmental potential of the embryo. The process of oocyte growth and gene transcription leads to increased synthesis and accumulation of mRNA and protein in the oocyte [6, 7]. Notably, transcription is silenced in germinal vesicle (GV) stage oocytes, that is, oocytes at the diplotene stage during the first meiotic prophase, when their size approaches that of a mature oocyte [7–9]. Silencing of gene transcription in oocytes is required for the recovery and completion of the first meiotic division [7–9]. Failure of GV oocytes to undergo transcriptional silencing has significantly negative impact on subsequent oocyte maturation, fertilization and embryonic development [10–12].

The transcriptional activity of oocytes is closely related to the morphology of the nucleus [11–13]. In mice, the chromatin conformation changes from non-surrounded nucleolus (NSN) to surrounded nucleolus (SN) as GV-stage oocytes grow [12–15]. Studies have shown that there is high transcription activity in NSN oocytes, while the transcription event in SN is silenced, demonstrating that the process of chromatin transition from NSN to SN is crucial for the generation of mature oocytes with normal functions [13–16]. The chromatin changes in human oocytes are similar to those in mice [14, 17], but due to the difficulty of sample acquisition, the process of chromatin changes in human oocytes is not well understood.

The related biological events during NSN-to-SN transition are complicated, including the regulation of gene expression, protein translation, and various biological metabolic pathways. Regulation of both transcription factors (TFs) and transposable elements (TEs) are involved in the regulation of gene expression [18, 19]. Studies showed that epigenetic modification factors also play an important role in the regulation of the overall chromatin opening state and specific gene expression by changing the chromatin structure of oocytes [20–24]. The effects of important modifications including histone acetylation and lactylation on SN formation are still unknow [25–30]. In addition, the related biochemical metabolism, protein kinase regulatory pathways, and metabolites changes in oocytes all participate in the regulation of NSN-to-SN transition [30–34]. Therefore, it is meaningful to identify the factors regulating the transition process.

Herein, to investigate the regulatory mechanism of chromatin transition in human oocytes, we collected donated human oocytes to discriminate NSN and SN oocytes by DNA staining, and analyzed their transcriptional profiles subsequently. Our result supports that the transcriptional activity was decreased in human SN oocytes, and analysis on differentially expressed gene showed that epigenetic regulation, protein kinase regulatory network and transposable elements may play key roles in NSN-to-SN transition of human oocytes.

## Results

### Establishment of transcriptional profiles of human NSN and SN oocytes

**Fig. 1:**
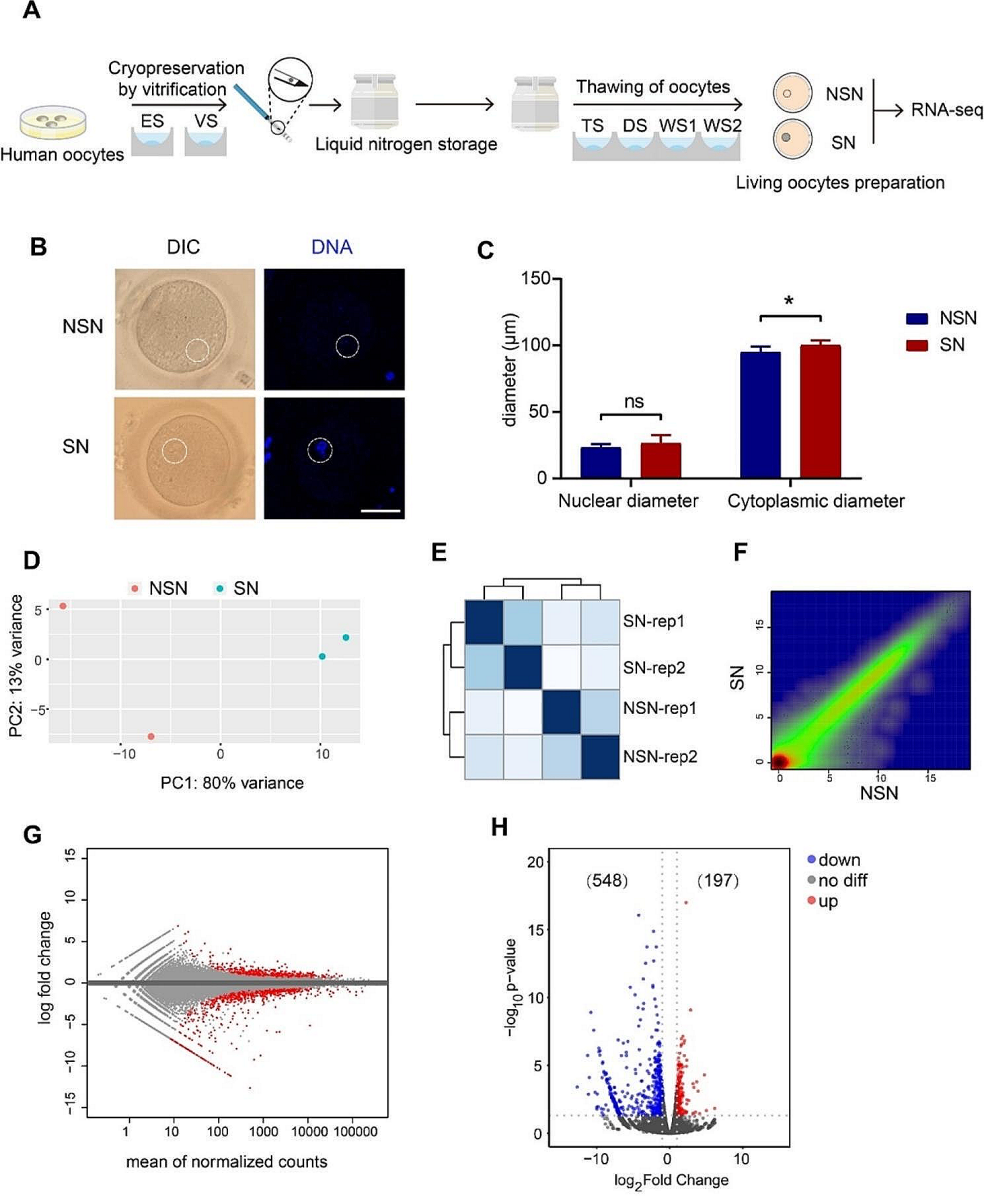
The establishment of transcriptional profiles of human NSN and SN oocytes. (**A**) Schematic illustration of the cryopreservation and thawing of human oocytes. (**B**) DIC and DNA staining of human NSN/SN oocytes. Bar, 50 μm. (**C**) Bar graph showing the nuclear and cytoplasmic diameters of human NSN/SN oocytes. (**D**) PCA of transcriptome of human NSN/SN oocytes. (**E**) Hierarchical clustering of human NSN and SN oocyte transcriptome. (**F**) Density scatter plot of human NSN and SN oocyte transcriptome. (**G**) MA plot of differentially expressed genes in human NSN and SN oocytes. (**H**) Volcano diagram showing differentially expressed genes of human NSN and SN oocytes

To obtain two types of human oocytes with different chromatin configurations, we collected donated human oocytes and cryopreserved them by vitrification for short term storage (Fig. 1A). After thawing and examining the oocytes by Hoechst staining, we were able to discriminate the two types of chromatin configurations of human oocytes by confocal microscopy (Fig. 1B). We firstly measured the longest diameter of the nucleus as the “nuclear diameter”, and also identified the longest cytoplasmic diameter as the “cytoplasmic diameter”. We only noticed that the cytoplasmic diameter of human SN oocytes was longer than that of NSN oocytes, indicating size differences of these two types of oocytes, and this result was consistent with that in mice (Fig. 1C).

In order to map the transcription profiles of NSN and SN oocytes, the two types of oocytes were collected separately and transcriptome profiling was identified to reveal the differences in gene expression. PCA and hierarchical clustering analysis demonstrated that there was significant difference in the transcriptome of the two cell types (Fig. 1D-E), indicating the reliability of our sequencing data. Scatterplot showing expression of individual genes demonstrated that there were both up-regulated and down-regulated transcripts in SN oocytes relative to NSN oocytes (Fig. 1F). Normally, the transition from the NSN to the SN state in the oocyte is characterized by a gradual decrease in transcriptional activity until transcription is silenced and the oocyte is ready to enter meiosis [17, 35]. The most of the differentially expressed genes in SN oocytes were down-regulated genes (548/745) compared with NSN (Fig. 1G-H), indicating that the transcriptional activity was decreased in SN oocytes. Interestingly, we noticed that a small number of genes were still up-regulated in SN oocytes (197/745), and these genes may have transcriptional activity and play an important role in maintaining the structure of SN oocytes (Fig. 1G-H).

### Inhibition of lactate dehydrogenase B (LDHB) promoted NSN-to-SN oocyte transition

**Fig. 2:**
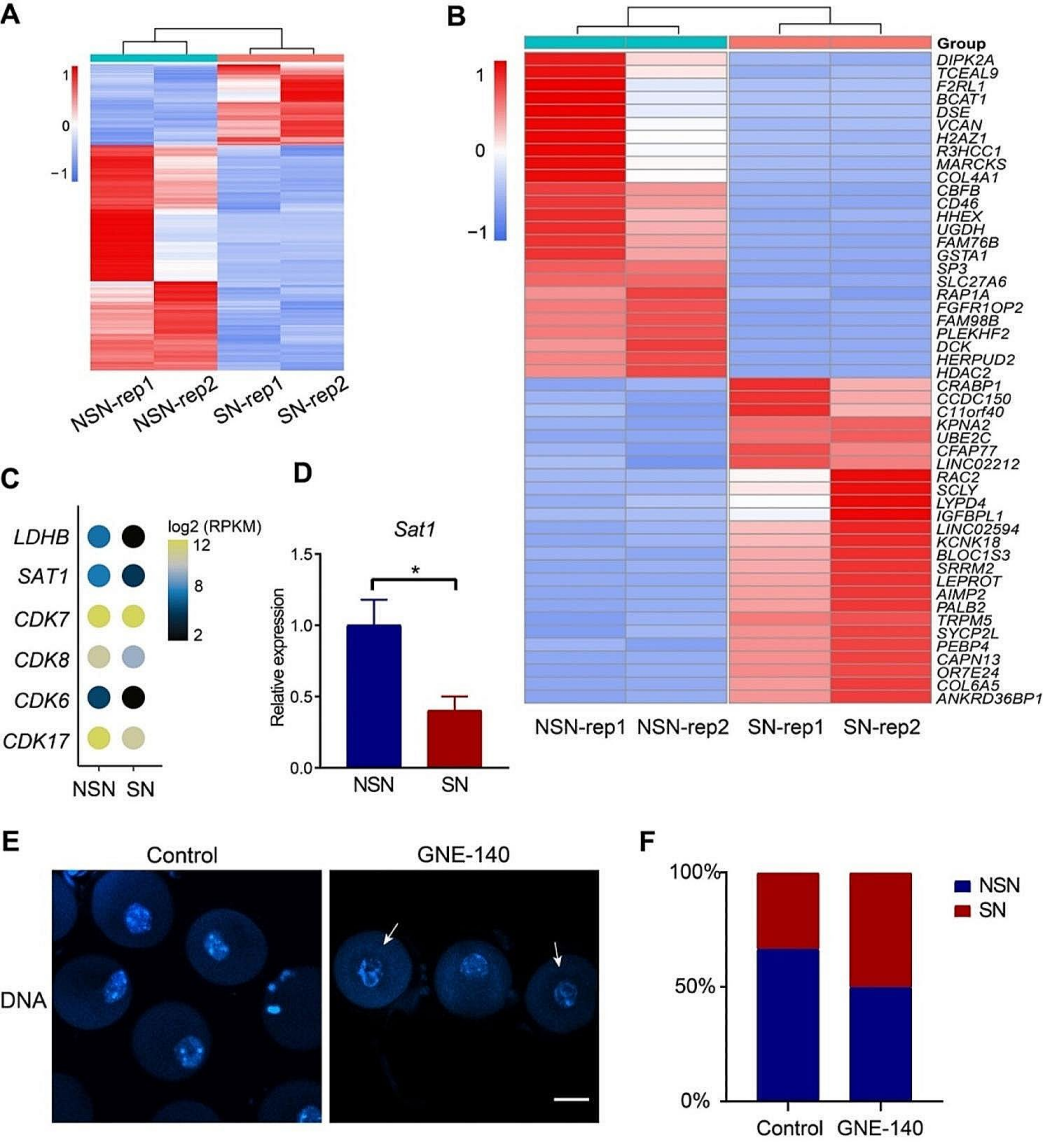
Identification of differentially expressed genes between NSN and SN oocytes. (**A**) Heatmap showing all differentially expressed genes in human NSN/SN oocytes. Rep1/2 represents sample replicates. (**B**) Heatmap showing the top 25 up-regulated genes and top 25 down-regulated genes in human NSN/SN oocytes. (**C**) Dots plot showing the differentially expressed genes in human NSN/SN oocytes. (**D**) Bar plot showing the relative expression of *Sat1* in mouse NSN/SN oocytes (*n* = 3). Data are presented as means ± SD. Two-tailed student’s t-test was used to calculate *p* values. ∗ *p* < 0.05. (**E**) Representative DNA staining image of mouse NSN/SN oocytes. Arrows indicate SN oocytes. Bar, 50 μm. (**F**) Proportions of mouse NSN/SN oocytes in GNE-140 treatment group and control group

Differentially expressed genes were identified by DESeq2 (Fig. 2A), and top 25 up-regulated or down-regulated genes showed that different subsets of genes were enriched in the two types of human oocytes (Fig. 2B-C). Due to the difficulty of obtaining human GV oocyte samples, mouse GV oocytes were used to verify the results. Spermidine/spermine N1-acetyltransferase 1 (SAT1), a kind of rate-controlling enzyme which regulates the catabolism of polyamine catabolism [36], was found to be highly expressed in human NSN oocytes by RNA-seq (Fig. 2C). RT-qPCR result using mouse NSN and SN oocytes on biased *Sat1* expression was consistent with RNA-seq result of human oocytes (Fig. 2D).

LDHB plays an important role in the conversion of lactate to pyruvate, and maintaining the level of lactate is crucial for early embryos [30, 31]. Studies have shown that early embryonic development of mice was severely arrested after LDHB inhibition, accompanied by abnormal gene expression and histone modification [30]. Particularly, we found that *LDHB* transcript level was higher in human NSN oocytes but almost no expression in human SN oocytes (Fig. 2C). To investigate the factors influencing the configuration transition from NSN to SN, we treated mouse oocytes with GNE-140, an inhibitor of LDHB, to detect the proportion of SN oocytes. The result showed that the proportion of SN oocytes upon GNE-140 treatment was increased compared to the control group (Fig. 2E-F), indicating that LDHB inhibition drove formation of SN configuration, probably through controlling lactate level.

Taken together, we identified differential gene profiles between human NSN and SN oocytes. The decreased expression level of *LDHB* in human NSN-to-SN transition implied that LDHB depletion contributes to the formation of SN oocytes, and this function was confirmed in mouse oocytes. This result suggests that LDHB inhibition may be applied to in vitro maturation of human oocytes to improve efficiency of assisted reproductive technology.

### Differentially expressed genes indicated potential molecular mechanisms involved in human NSN-to-SN oocyte transition

**Fig. 3:**
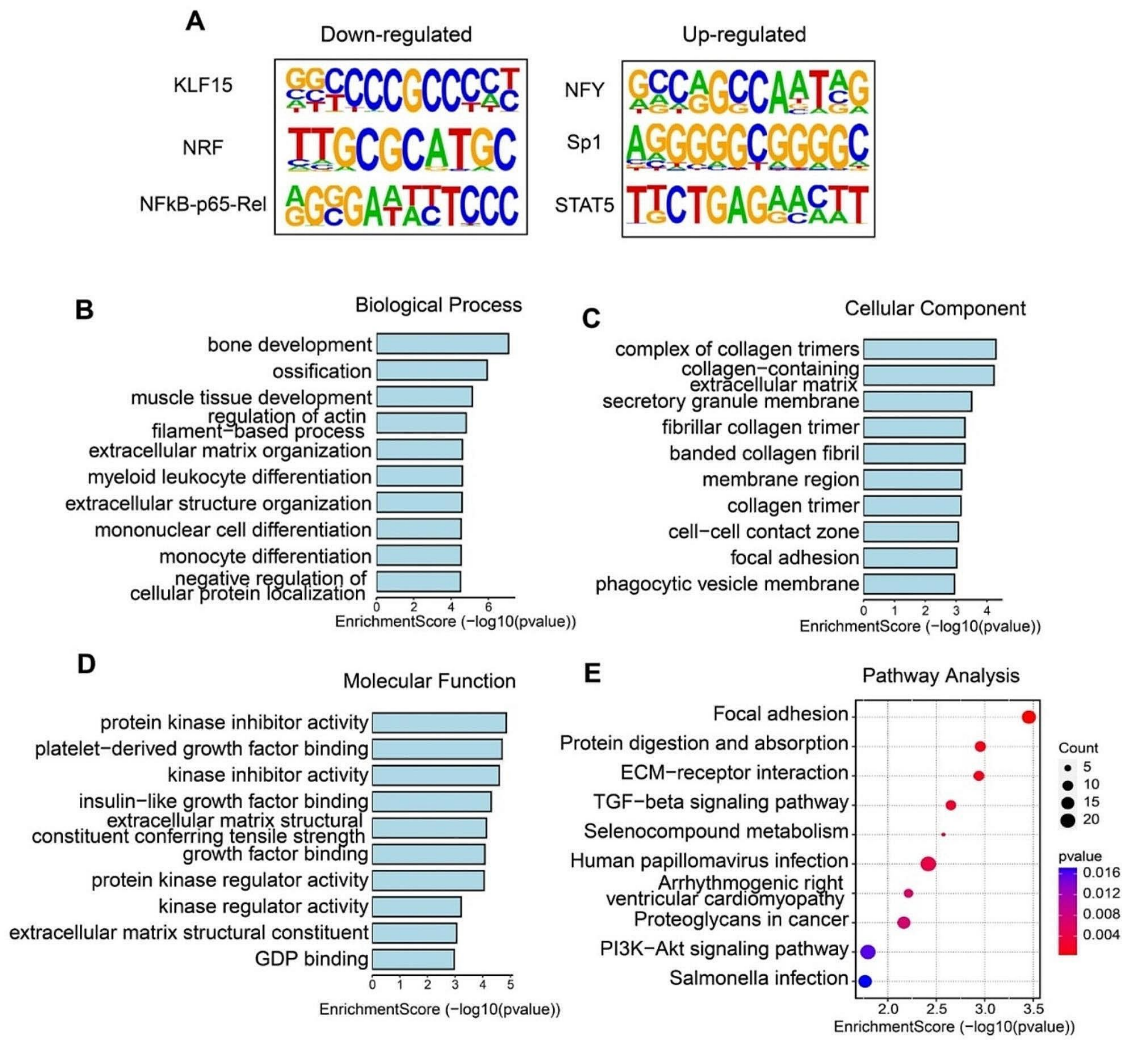
The function enrichment analysis of differentially expressed genes in human NSN/SN oocytes. (**A**) Motif analysis of promoters of up/down-regulated genes by HOMER algorithm. (**B**-**D**) GO analysis of differential expressed genes between human NSN and SN oocytes. (**E**) KEGG analysis of differential expressed genes between human NSN and SN oocytes

For differentially expressed genes, motif analysis showed that KLF15, NFY and other transcription factors were enriched (Fig. 3A). Unexpectedly, the vital roles of Krüppel-like factor 17 (KLF17) in activating genes and promoting embryonic development were indicated recently [37]. While, KLF15, the factor from the same family, may play important roles during oocyte maturation.

H2AZ1 (H2A.Z Variant Histone 1) is a well-known histone variant involved in histone acetylation and gene regulation [25–29]. *H2AZ1* gene was found to be down-regulated in human SN oocytes (Fig. 2B), indicating its downregulation may be involved in genome silencing in human SN oocytes. Previous studies reported that organization of chromatin morphology in oocytes impacts meiosis progress [34, 38]. The cyclin-dependent kinases (CDKs) are the core molecules in the cell cycle regulation mechanism, meanwhile, the role of CDK in the regulation of gene transcription through phosphorylated RNA polymerase II was also identified recently [32–34]. In our analysis, several CDKs showed higher expression levels in human NSN oocytes, in agreement with higher transcription activity of NSN oocytes (Fig. 2C). Actually, other protein kinase-related genes such as *DIPK2A* and *MARCKS* were also found to be highly expressed in human NSN oocytes (Fig. 2B), implicating tuning down of protein kinase regulatory network during NSN-to-SN transition.

The results of Gene Ontology (GO) enrichment analysis for biological process, cellular component and molecular function showed enriched biological events related to differentially expressed genes, and the regulation of protein kinases was also found (Fig. 3B-E). Kyoto Encyclopedia of Genes and Genomes (KEGG) is a kind of analysis that uses databases to systematically analyze gene functions and related metabolic pathways [39], and TGF-beta and PI3K-Akt signal pathways were enriched (Fig. 3E and S1), which are closely related to protein activity, protein transport, post-transcriptional modification and other vital cellular events [40, 41]. Above results demonstrated that several biological events are involved in the process of chromatin configuration transition and provided clues for further investigation on key regulators involved in the regulation of human oocyte maturation.

Several up-regulated genes in human SN oocytes were also demonstrated (Fig. 2B). For example, the up-regulation of *UBE2C* (ubiquitin conjugating enzyme E2 C), a member of the protein degradation pathway genes [42], suggested involvement of ubiquitination activity during the process of chromatin configuration transition. Degradation of maternal proteins in oocytes is regarded to be beneficial to the normal development of early embryos [43], and our results were consistent with this view.

Taken together, we found that the histone modifications, protein kinases, and the *CDK* family genes may play important roles in NSN-to-SN transition of human oocytes. Involvement of TGF-beta and PI3K-Akt signaling pathway which may regulate chromatin morphology in human oocytes were also revealed. Interestingly, the up-regulation of ubiquitin-related protein UBE2C in SN oocytes implies the activity of protein degradation during human oocyte maturation.

### The expression of LINE and SINE was significantly different between human NSN and SN oocytes

**Fig. 4:**
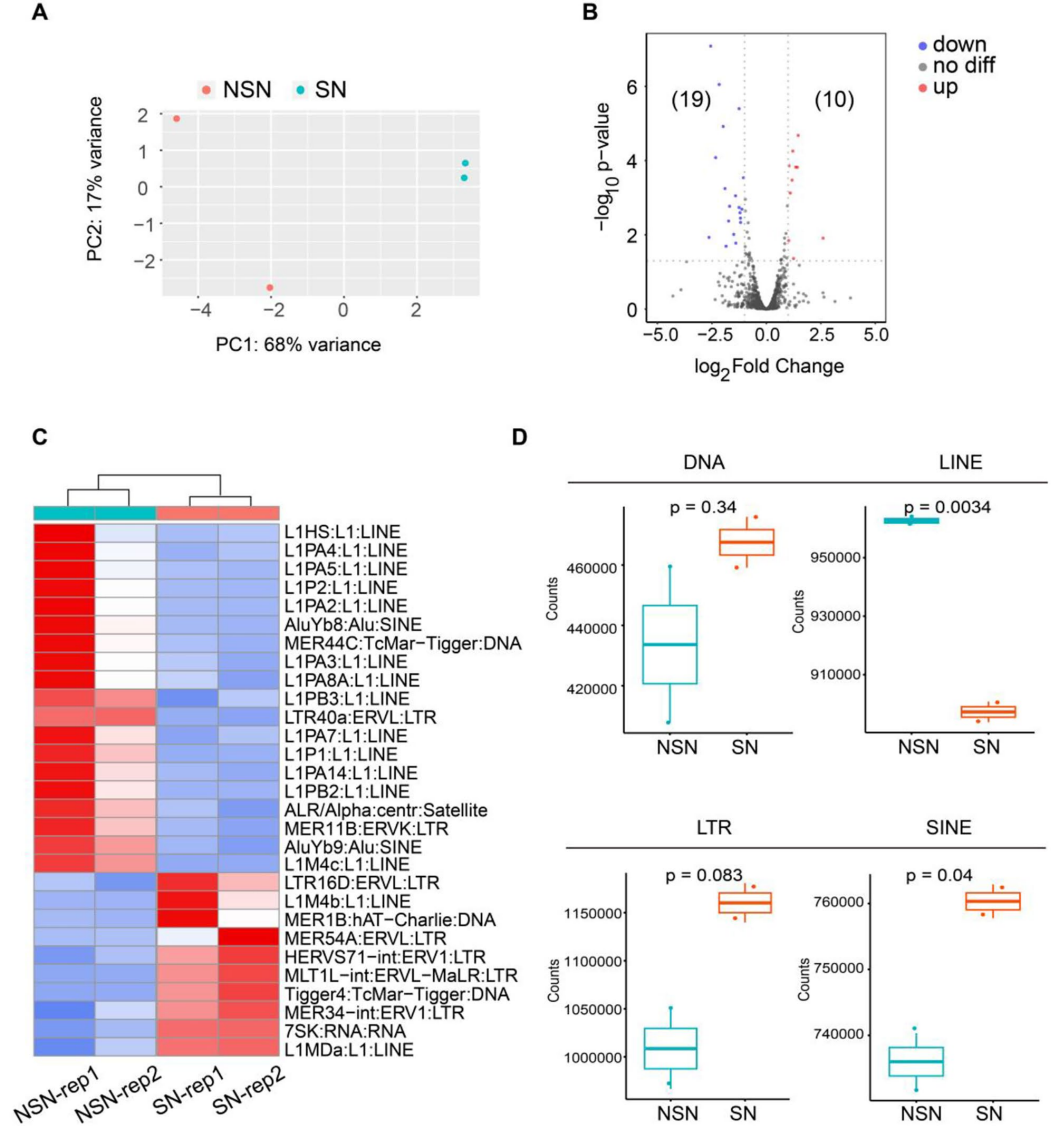
TEs were differentially enriched in human NSN and SN oocytes. (**A**) PCA of TE enrichment of human NSN and SN oocytes. (**B**) Volcano diagram showing differentially expressed TE transcripts between human NSN and SN oocytes. (**C**) Heatmap showing the all differentially expressed TE transcripts between human NSN and SN oocytes. (**D**) Box plot comparing the counts of differentially expressed TEs in DNA, LINE, LTR and SINE families

Transposable element (TE) has the potential to disrupt the structure of genes, and is considered to be one of the causes of chromosome insertion, deletion, and transposition [18, 19]. To further analyze the differences in gene expression between human NSN and SN oocytes, we investigated TE enrichment in the two types of oocytes and performed PCA analysis (Fig. 4A). We found that several TE family genes were differentially enriched in human NSN and SN oocytes (Fig. 4B). It showed that most of the down-regulated TEs belonged to long interspersed nuclear element (LINE) family (Fig. 4C). We also calculated normalized counts of TE subfamilies for comparison and found that the expression of LINE family elements was significantly reduced in human SN oocytes, suggesting that silencing of LINE plays important roles in genome silencing and maintenance of genome stability (Fig. 4D). Interestingly, the slight activation of short interspersed nuclear element (SINE) implied establishment of a new chromatin organization pattern in human SN oocytes (Fig. 4D). Meanwhile, we didn’t notice expression changes of DNA transposons or LTR elements. These results suggested that retrotransposon LINE and SINE may play important roles in the regulation of gene activity and chromatin configuration in GV oocytes.

## Discussion

As the proportion of female infertility increases throughout the world, more and more people accept and adopt ART to obtain offspring, and the acquisition of high-quality oocytes is an indispensable step in ART [1–3]. During oocyte development, chromatin undergoes a conformational change, and the transition from NSN to SN accompanied by transcriptional silencing is a crucial step [13–15]. In this study, we collected donated human oocytes, classified them into NSN and SN oocytes for transcriptome analysis. Our analysis identified potential regulatory mechanisms of chromatin configuration control.

Generally, high transcriptional activity is present in NSN oocytes, whereas the transcription activity of SN oocytes is silent. By low-input RNA-seq, we found that there were many down-regulated genes in human SN oocytes compared with NSN oocytes, which implied the silencing process of gene transcription. One of these genes is *LDHB*, which plays an important role in the maintenance of lactate. Our experiments using mouse oocytes showed that the inhibition of LDHB with GNE-140 promoted the formation of SN oocytes, indicating that LDHB inhibitor may be a promising drug to improve in vitro human oocyte maturation.

Epigenetic modifiers like H2A.Z1 are well known in regulating transcriptional events. Significant decrease of expression of H2A.Z1 in human SN oocytes hints that its inhibition may facilitate chromatin transition to obtain matured SN oocytes thereby increase the efficiency of in vitro maturation technique.

It is noteworthy to mention that the expression level of protein kinases including CDKs are high in human NSN oocytes, reflecting the high metabolic activity of oocytes at this stage. PI3K-Akt signal pathway is involved in intracellular signal transduction pathways and promotes cell metabolism and proliferation [41], while TGF-beta signaling pathway participates in the regulation of transcription level through protein interaction and protein post-translational modification [40]. Both signaling pathways were found to be enriched in differentially expressed gene pathways, and they act as key metabolic pathways in oocytes to jointly accomplish various biochemical activities under the high transcriptional activity of human NSN oocytes.

Our results suggest that degradation-related genes have elevated transcriptional activity during the transition from human NSN to SN oocytes to facilitate the degradation of redundant maternal products for subsequent embryo development, such as *UBE2C*. Activation of UBE2C-mediated ubiquitination in human NSN may provide a new approach to study human oocyte maturation in vitro. We also found that the abundance of TE elements was different between human NSN and SN oocytes. LINE silencing suggested a way for oocytes to avoid damaging genome stability during ovarian storage of silent oocytes, while SINE activation implied a new regulatory pathway for transposable elements to be established to prepare for later development.

## Conclusions

In summary, we have collected and successfully mapped the transcription profiles of human NSN and SN oocytes to investigate the processes of chromatin transition and transcriptional silencing. Our results revealed the regulatory network from human NSN to SN oocytes and provided an insight into in vitro maturation and acquisition of high-quality human oocytes in ART.

## Methods

### Human oocytes preparation

Human GV oocytes were obtained from Center of Reproductive Medicine, Tongji Medical College, Huazhong University of Science and Technology. Human oocytes were derived from patients with assisted reproduction needs, who had different kinds of reproductive problems. Patients were treated with hormone drugs such as gonadotropin-releasing hormone agonists to promote the growth and maturation of follicles, and then oocytes were collected with the assistance of ultrasonic apparatus using an oocyte retrieval needle. One to two GV oocytes were collected from each patient. The donated oocytes were collected in liquid nitrogen followed by protocol of vitrification freezing kit (Kitatazo, Japan). A total of 55 GV oocytes from patients were included in this study, and each oocyte group was pooled sample. Generally, oocytes were stored in Equilibrium Solution vial (ES) for 5 to 10 min, and transferred to vitrification Solution vial (VS) for 40–60 s. Then oocytes were stored at Cryotop (Kitatazo, Japan) in liquid nitrogen. For oocyte thawing, we obtained oocytes from Cryotop, and placed them into 37℃ Thawing Solution (TS) for 1 min. Next, oocytes were transferred to Diluent Solution (DS) for 3 min, Washing Solution 1(WS1) for 5 min, and Washing Solution 2 (WS2) for 5 min at room temperature with a Pasteur pipette.

### Mouse oocytes preparation

Adult ICR female mice were purchased from Bainte Biotechnology Company (Hubei, China). To collect GV oocytes, female mice were sacrificed by cervical dislocation, and the ovaries were removed and sliced to extract GV oocytes. GV oocytes were cultured in modified KSOM medium (95 mM NaCl, 2.5 mM KCl, 0.35 mM KH_2_PO_4_, 0.20 mM MgSO_4_, 25 mM NaHCO_3_, 1.71 mM CaCl_2_, 0.01 mM EDTA, 0.20 mM glucose, and 0.20 mM pyruvate) devoid of lactate, amino acid and BSA [30], supplemented with 2.5 μM milrinone (Sigma, USA).

### Ethics statement

Ethical approval for the human oocyte study was obtained by the CEIC (Ethics Committee for Clinical Research) of Reproductive Medicine Center, Tongji Medical College, Huazhong University of Science and Technology. All people included in the study gave informed consent.

Animal procedures were approved by the Institutional Animal Care and Use Committee of Tongji Medical College, Huazhong University of Science and Technology (IACUC Number 3028). Mice were housed in the specific pathogen-free facility of Huazhong University of Science and Technology. All experiments with mice were conducted ethically according to the Guide for the Care and Use of Laboratory Animal guidelines.

### Hoechst staining of GV oocytes

We totally obtained two groups of pooled human GV oocytes (group 1 for NSN-rep1 and SN-rep1; group 2 for NSN-rep2 and SN-rep2). Each group was independently vitrified and thawed, and GV oocytes with normal morphology were cultured in G-1 medium (Vitrolife, 10,127, Sweden) with Hoechst 33,342 (10 μg/ml, Invitrogen, USA) for 1 h. Confocal microscopy was performed using laser scanning microscope (LSM780, Zeiss) to distinguish NSN and SN oocytes. *n* = 11 for NSN-rep1, *n* = 9 for SN-rep1, *n* = 12 for NSN-rep2, *n* = 13 for SN-rep2, and 10 oocytes were not used because of low quality or unclear chromatin configuration. For Hoechst staining of mouse GV oocytes, oocytes were cultured in modified KSOM medium with 10 μg/ml Hoechst 33,342 and 2.5 μM milrinone for 1 h, followed by confocal microscopy examination.

### GNE-140 treatment of mouse oocytes

Mouse GV oocytes were divided into two groups. 50–60 oocytes were collected in each group and cultured in culture medium for 24 h at 37℃. The culture medium of control group was modified KSOM medium with 2.5 μM milrinone. In the GNE-140 treatment group, the culture medium was modified KSOM medium with 2.5 μM milrinone and 10 μM GNE-140. After 24 h, the GV oocytes were stained by 10 μg/ml Hoechst 33,342 and examined by confocal microscopy (LSM780, Zeiss) to distinguish NSN and SN oocytes. GraphPad Prism (8.0.2) was used to draw bar graph.

### RNA-seq library generation and sequencing

A total of 5–10 human NSN or SN oocytes in each group were collected for RNA-seq library preparation. Generally, oocytes were collected in tubes with lysis component and ribonuclease inhibitor. Then amplification was carried out by the Smart-Seq2 method (Takara). Qualified libraries were loaded onto Illumina Hiseq platform for PE150 sequencing by Annoroad Gene Technology. For QC of RNA-seq data, see Table S1.

### High-throughput data analysis

Raw reads were processed with Trim Galore (v0.6.4) to remove adaptor sequences and poor-quality bases with ‘--q 20 --phred33 --stringency 5 --length 20 –paired’. Trimmed reads were aligned to the human reference genome (hg19) using STAR (v2.7.5b) with default settings. SAMtools (v1.3.1) was used to sort bam files by genomic coordination and make a bam file index. R package DESeq2 (v1.28.1) was used to obtain differentially expressed genes (DEGs) based on the reads count file obtained by STAR (v2.7.5b). Genes with an absolute log2FoldChange > 1 and P adjusted < 0.05 were considered as significant DEGs. Principal component analysis of RNA-seq was performed using the prcomp function in R. Correlation of different samples was calculated by multiBamSummary/multiBigwigSummary and plotCorrelation of the Python package deepTools (v3.5.1). GO and KEGG analysis were plotted by https://www.bioinformatics.com.cn, an online platform for data analysis and visualization. HOMER (v4.11) was used to analysis the motif for gene promoter region from individual clusters. Boxplot, scatterplot, volcano plot, heatmap were generated by ggplot2 in R (v3.3.2).

### RT-qPCR

For mouse oocyte RNA isolation, adult ICR female mice were used. The mice were sacrificed by cervical dislocation, and the ovaries were removed and sliced to extract GV oocytes. After DNA staining, 25–40 NSN or SN oocytes were collected into tubes with 500 μl TRIzol reagent (Sigma, USA) for 5 min. Then 200 μl of Chloroform (HONEYWELL, China) was added and thoroughly mixed, followed by centrifugation at 12,000 rpm in 4℃ for 10 min. The supernatant was removed and then mixed with 1 μl glycogen and 200 μl Isopropanol (HUSHI, China) at -20℃ overnight. After centrifugation at 12,000 rpm in 4℃ for 25 min, RNA solution was obtained by dissolving the white precipitate with ultrapure water.

The RNA solution of NSN/SN oocytes were used to collect cDNA according to the instruction of Hifair® II 1st Strand cDNA Synthesis SuperMix (Yeasen, China). The NSN/SN cDNA was then mixed with primers and SYBR according to the instructions in Hieff® qPCR SYBR Green Master Mix (High Rox Plus) (Yeasen, China), and the fluorescence quantification was performed using the ABI 7500 Real-Time PCR system (Applied Biosystems, USA). The Ct values obtained were standardized by the expression of *Gapdh*, and the relative expression of *Sat1* in NSN and SN mouse oocytes was compared. The primer sequences for *Sat1* are: forward 5’-GGCTAAATTTAAGATCCGTCCA-3’, reverse 5’-CATGTATTCATATTTAGCCAGTTCCTT-3’. The primer sequences for *Gapdh* are: forward 5’-TCTTCCAGGAGCGAGACCC-3’, reverse 5’-CGGAGATGATGACCCTTTT-3’. GraphPad Prism (8.0.2) was used to draw bar plots.

### Statistical analysis

Genes with |log2Foldchange| > 1 and adjusted *p* value < 0.05 were regarded as significantly differentially-expressed genes.

### Electronic supplementary material

Below is the link to the electronic supplementary material.

Supplementary Material 1

Supplementary Material 2

## Data Availability

The raw sequence data reported in this paper have been deposited in the Genome Sequence Archive in National Genomics Data Center, China National Center for Bioinformation/Beijing Institute of Genomics, Chinese Academy of Sciences (GSA-Human: HRA006194) that are publicly accessible at https://ngdc.cncb.ac.cn/gsa-human.
