# Supplementary figures and images for: The landscape of transcriptional profiles in human oocytes with different chromatin configurations
Source: J Ovarian Res. 2024 May 10;17:99. doi: 10.1186/s13048-024-01431-2 (PMC11088011; doi:10.1186/s13048-024-01431-2)

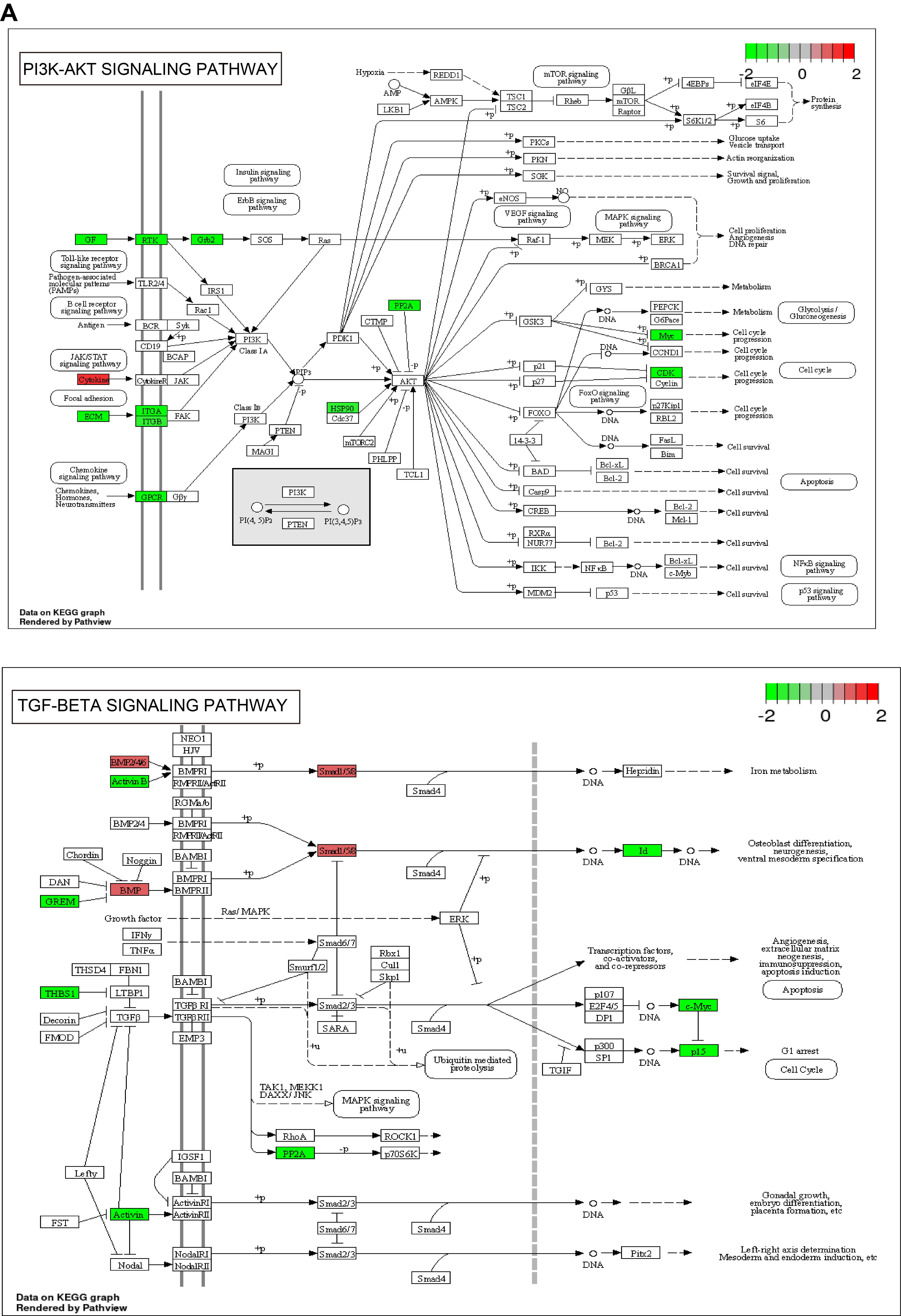


Figure S1. (A) The regulation networks of PI3K-Akt and TGF-beta signal pathways.

Supplement: Supplementary file 2 — Supplementary Material 2 [file 13048_2024_1431_MOESM2_ESM.docx]
